# Orbito-sinal foreign body with floor fracture: an unusual presentation

**DOI:** 10.3205/oc000159

**Published:** 2020-08-06

**Authors:** Narendra Patidar, Saket Agrawal, Rukmendra Pratap Singh, Prerana Phadnis

**Affiliations:** 1 Department of Orbit and Oculoplasty, Sadguru Netra Chikitsalaya, Shri Sadguru Seva Sangh Trust, Jankikund, Chitrakoot, Satna, Madhya Pradesh, India

**Keywords:** orbito-sinal foreign body, floor fracture, fracture repair

## Abstract

Wooden foreign bodies penetrating through the orbit into paranasal sinuses are rare. We report a case of a young male who complained of double vision, pain and redness after a fall from a tree. There was no external wound over periocular skin. The clinical and radiological examination was suggestive of an inferior orbito-sinal wooden foreign body with floor fracture, which was managed by surgical removal of the foreign body and orbital floor fracture repair using a silicon sheet in a single sitting.

## Introduction

Orbital foreign bodies may present with varying clinical features and are often difficult to diagnose. A computed tomography (CT) scan is a useful tool in cases of orbital trauma. Wooden foreign bodies present a diagnostic challenge as they may easily be missed on imaging. They appear as low intensity in the early period, and attenuation increases over time as they absorb water [1]. Foreign body removal is also a challenge as they are fragile and tend to get broken during surgery, which may result in incomplete removal [2]. Herein, we report a case of a wooden foreign body in the inferior orbit extending to the maxillary sinus through an orbital floor fracture.

## Case description

A 17-year-old male presented to the Department of Orbit and Oculoplasty, Sadguru Netra Chikitsalaya one week after he sustained a fall from a tree. He reported double vision, pain and redness in the right eye. CDVA was 20/20 in both eyes. IOP by the non-contact tonometry was 16 mm Hg and 14 mm Hg in the right and the left eye respectively.

**Figure 1:**
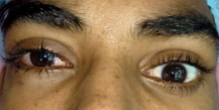
Superior displacement of the globe with no external scar

**Figure 2:**
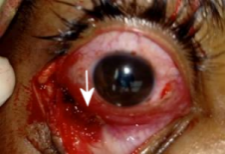
Intraoperative photograph showing the entry site in the inferior fornix (arrow)

On examination, the patient had right-sided superior displacement of the globe and limitation of movement in all gazes. Lower lid edema was present, but the periocular skin had no external scar (Figure 1 (Fig. 1)). 2 mm proptosis was present on the right side. On further evaluation, one end of a wooden stick was noticed in the inferior fornix with congestion of inferior fornicial conjunctiva and granulation tissue around the entry wound (Figure 2 (Fig. 2)). Fundus examination showed elevation in the inferior retina and few superficial haemorrhages, which was suggestive of mass effect. The rest of the anterior segment examination was normal.

**Figure 3:**
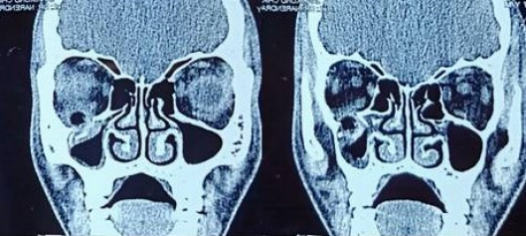
CT scan showing the foreign body in the inferior orbit extending to the maxillary sinus through floor fracture

Computed tomography revealed a well-defined, linear, hypodense, air-filled tract suggestive of a foreign body in the inferior orbit extending to the maxillary sinus (Figure 3 (Fig. 3)). An undisplaced orbital floor fracture was noted as well. In the orbital blowout fracture, a CT scan also showed orbital floor disruption. However, since this patient had proptosis and superior displacement of the globe and an embedded foreign body, it was an unusual presentation of a floor fracture. The patient was started on oral antibiotics, analgesics and antifungal for a week. Once inflammation was reduced, the patient was taken for surgical removal of the foreign body and orbital floor fracture.

**Figure 4:**
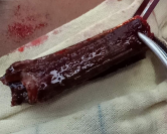
Wooden foreign body measuring 4 cm x 1 cm

Under general anaesthesia, lateral canthotomy was done, and a twig measuring around 4 cm x 1 cm was removed (Figure 4 (Fig. 4)). Then inferior orbitotomy through a conjunctival incision was done, and the bony defect was sealed with a silicone sheet (Attachment 1). Layered closure of the wound was performed, followed by canthoplasty.

**Figure 5:**
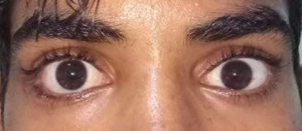
Postoperative photograph after 4 months

The patient received antibiotics (inj. cefotaxime 1 g IV BD), steroids (inj. dexamethasone 8 mg IM OD), oral non-steroidal anti-inflammatory drugs and antacids for 3 days postoperatively, and was discharged on oral antibiotics (tablet ampicillin and cloxacillin 500 mg) for another 5 days. The postoperative recovery was normal. The patient was asymptomatic and had only minimal residual restriction and diplopia in up gaze on 4-month follow-up (Figure 5 (Fig. 5)). The mass effect in the retina also subsided.

## Discussion

Trauma due to foreign bodies may have varied presentations. Imaging plays a crucial role in ascertaining the presence or absence of a foreign body, or, as in our case, assessing the extent of penetration.

Once the exact location of the foreign body has been assessed, the surgical procedure can be planned accordingly. Jagannathan et al. [3] reported a metallic foreign body which was lodged in the infratemporal fossa, the maxillary antrum, and the floor of the orbit, and was removed through the maxillary antrum. Simha et al. [4] reported a case of an orbito-sinal foreign body which pierced the orbital floor posteriorly close to the orbital apex. They performed a combined approach – sublabial and inferomedial orbitotomy. We chose a conjunctival approach alone, as in our case the anterior end of the foreign body was visible through the fornix.

## Conclusion

Size, location and type of the foreign body are the most crucial factors in deciding on the surgical approach. Co-existing morbidities such as orbital fracture also have to be addressed. Proper surgical planning results in a good outcome with minimum tissue damage. Adequate antibiotic coverage is paramount to prevent infections to important ocular structures.

## Notes

### Competing interests

The authors declare that they have no competing interests.

### Informed consent

The patient has given informed consent for the publication of this case report.

## Supplementary Material

Video: Removal of wooden foreign body by inferior orbitotomy through a conjunctival incision
